# The Association Between Alveolar Dead Space Fraction and Mortality in Pediatric Acute Respiratory Distress Syndrome: A Prospective Cohort Study

**DOI:** 10.3389/fped.2022.814484

**Published:** 2022-02-28

**Authors:** Sheow Boon Oh, Apollo Aguilan, Herng Lee Tan, Yi-Jyun Ma, Rehena Sultana, Jan Hau Lee, Judith Ju Ming Wong

**Affiliations:** ^1^Lee Kong Chian School of Medicine, Singapore, Singapore; ^2^Children's Intensive Care Unit, Department of Pediatric Subspecialties, KK Women's and Children's Hospital, Singapore, Singapore; ^3^Center for Quantitative Medicine, Duke-NUS Medical School, Singapore, Singapore; ^4^Duke-NUS Medical School, Singapore, Singapore

**Keywords:** acute respiratory distress syndrome, acute lung injury, pediatrics, artificial respiration, carbon dioxide, blood gas analysis, mortality

## Abstract

**Background:**

Alveolar dead-space fraction (AVDSF), the volume of alveolar gas that does not participate in gas exchange, has been reported to predict mortality and morbidity in adults with acute respiratory distress syndrome (ARDS). This study aims to characterize AVDSF in patients with pediatric ARDS (PARDS), to determine its association with clinical outcomes and examine the validity of a previously studied cutoff (AVDSF > 0.25).

**Methods:**

This was a prospective cohort study performed in the setting of a lung protective mechanical ventilation protocol. AVDSF was calculated by the equation: AVDSF = [partial pressure of arterial carbon dioxide (P_a_CO_2_) – end tidal carbon dioxide (etCO_2_)]/P_a_CO_2_. Receiver operating curve and Youden index were used to identify an AVDSF cutoff associated with mortality, which characterized “high or low AVDSF” groups. Correlation between AVDSF and clinical indices of severity were determined [including daily oxygenation index (OI), admission Pediatric Index of Mortality 2 (PIM 2) and Pediatric Logistic Organ Dysfunction (PELOD) scores]. The primary outcome, mortality in PARDS patients, was compared between the high and low AVDSF groups and analyzed in a multivariable logistic regression adjusting for inotrope use and PIM 2 score. Secondary outcomes included 28-day ventilator-free (VFD) and intensive care unit-free (IFD) days.

**Results:**

Sixty-nine PARDS patients with a median (interquartile range) age of 4.5 (0.8, 10.6) years were included in this analysis. Daily AVDSF correlated with daily OI (*R*^2^ = 0.10; *p* < 0.001). Mean AVDSF over the first 7 days of PARDS correlated with PIM 2 (*R*^2^ = 0.10; *p* = 0.010) and PELOD (*R*^2^ = 0.12; *p* = 0.004) scores. The greatest area under the curve identified an AVDSF cutoff of 0.22, which was close to the previously suggested 0.25. The high AVDSF group had higher mortality [7/19 (36.8%) vs. 5/50 (10.0%); *p* = 0.009] and lower VFD [2 (0, 18) vs. 21 (15, 24); *p* = 0.007] and IFD [0 (0, 16) vs. 16 (5, 21); *p* = 0.013]. In the multivariable model, being in the high AVDSF group [adjusted odds ratio 4.67 (95% CI: 1.12, 19.39)] was significantly associated with mortality.

**Conclusions:**

High AVDSF was independently associated with increased mortality and decreased VFD and IFD. AVDSF may be complementary to oxygenation indices in risk stratifying PARDS and warrant further study.

## Introduction

Acute respiratory distress syndrome (ARDS), the most severe form of acute hypoxic respiratory failure, has significant mortality rates in both adult and pediatric populations (1, 2). It is characterized by pulmonary edema and leukocyte infiltration not completely attributable to cardiac failure (3). At the cellular level, there is endothelial injury with microscopic thrombi formation, fibrocellular intimal proliferation, and eventual microvascular collapse (4). The resultant decreased perfusion causes dead space formation. The dead space fraction is the ratio of the dead space (V_D_) ventilation to tidal volume (V_T_). Existing literature in adult ARDS suggests that V_D_/V_T_ was a predictor of mortality (5, 6). An observational study of adults with ARDS demonstrated that an increase in dead space was independently associated with mortality (5). Another retrospective analysis involving adult ARDS patients demonstrated a direct correlation between computed tomography scan defined lung inhomogeneities with physiologic dead space (7). In effect, the current literature in adult ARDS seems to suggest an association between dead space fraction and both mortality and morbidity.

Thrombotic/thromboembolic events occurring in the adult pulmonary microcirculation of patients with ARDS is well-described (8). Although biomarkers of endothelial injury (e.g., thrombomodulin, von Willebrand factor antigen, angiopoietin-2) in pediatric ARDS (PARDS) have been described (9–11), descriptions of actual thromboses in PARDS are sparse (12). A retrospective analysis of 95 children with acute hypoxemic respiratory failure demonstrated a correlation between AVDSF and oxygenation indices, as well as an association between AVDSF and mortality (13). This study proposed that AVDSF is a marker of multi-aspect lung dysfunction rather than an isolated marker of pulmonary vasculature collapse (13). Children on mechanical ventilation (MV) showed a significant difference between end-tidal carbon dioxide (etCO_2_) and arterial partial pressure of carbon dioxide (P_a_CO_2_) when arterial-alveolar oxygen ratio fell, suggesting that AVDSF indicated impairment of oxygenation (14). Large cardiopulmonary shunts (e.g., in pulmonary hypoxic vasoconstriction) have also been described to contribute to increased AVDSF by a greater increase in P_a_CO_2_ than alveolar partial pressure of carbon dioxide (P_A_CO_2_) (15). Taken together, these observations demonstrate that AVDSF reflects pulmonary pathology in many regards.

Current gaps in literature involve the sparsity of data, validation of previous studies, and lack of studies based on the relatively recent Pediatric Acute Lung Injury Consensus Conference (PALICC) definition of PARDS (16). A single-center observational study in PARDS patients determined that an initial AVDSF >0.25 was associated with increased mortality and morbidity (17). However, this study did not assess AVDSF beyond 24 h after PARDS onset and was susceptible to variations in ventilator management. These findings and cutoff have also not been validated in other cohorts. Our study aims to add to the literature by (1) characterizing the AVDSF trend during the course of PARDS, (2) establishing the association of AVDSF on clinical outcomes, and (3) examining the validity of the aforementioned AVDSF cutoff.

## Materials and Methods

### Patient Selection and Study Design

This study was conducted in KK Women's and Children's Hospital, Singapore, a single-center, 16-bedded, multidisciplinary pediatric intensive care unit (PICU) over June 2018 to July 2021. This was a prospective cohort study and included data from the intervention arm of a lung protective MV study (18). In brief, all consecutive patients fulfilling the PALICC criteria for PARDS and providing informed consent were recruited into this study (16). etCO_2_ monitoring was routinely implemented in all mechanically ventilated patients in our center since June 2018. All patients were ventilated on a lung protective ventilation protocol (18) (Supplementary Table 1). This study was approved by the SingHealth Centralized Institutional Review Board (CIRB reference number 3076/2017/E). Reporting was in accordance with the Strengthening the Reporting of Observational Studies in Epidemiology (STROBE) guidelines (19) (Supplementary Table 2).

### Data Collection

Demographic and clinical data were collected, including admission Pediatric Index of Mortality (PIM) 2 and Pediatric Logistic Organ Dysfunction (PELOD) scores (20, 21). Detailed ventilation data were collected, corresponding to routine blood gas measurements at 0,600–0,800 h daily for the first 7 days of PARDS, which in turn was used for calculating daily AVDSF and OI. This included the etCO_2_ corresponding to the measured P_a_CO_2_ if the patient was ventilated on a conventional mode of ventilation [pressure-controlled (PC) and volume-controlled (VC) ventilation]. In general, etCO_2_ and P_a_CO_2_ were recorded simultaneously—when this was not possible, a maximum time discrepancy of no more than 30 min was accepted. etCO_2_ monitoring was unavailable when alternative modes of ventilation such as high-frequency oscillatory ventilation (HFOV) were used. Both mainstream and side stream capnography were used; The IntelliVue MX800 monitor (Philips Medical Systems, Andover, MA) was used for etCO_2_ monitoring. The mainstream etCO2 cable used was the Capnostat M2501A (Philips Medical Systems, Andover, MA), together with the M2533A airway adapter (Philips Medical Systems, Andover, MA). The side stream etCO2 sampling line used was the Microstream VitaLine H Set (Medtronic Covidien, Minneapolis, MN).

### Definitions and Calculations

Dead space (V_D_) is the portion of each tidal volume that does not take part in gas exchange and includes anatomical (part of airways that do not contribute to gas exchange) and alveolar/physiologic dead space (alveoli which are well-ventilated but poorly perfused) (22). V_D_ contributes to ventilation/perfusion mismatch, one of the main mechanisms involved in ARDS pathophysiology (23). Given the challenges of measuring the partial pressure of alveolar carbon dioxide (P_A_CO_2_), partial pressure of arterial carbon dioxide is used instead (P_a_CO_2_). Volumetric capnography measures the mean breath-by-breath expired volume of carbon dioxide; however, this method is tedious and requires specialized equipment (24). Instead, we used time-based capnography to measure etCO_2_, which was routinely implemented for mechanically ventilated children in our center and shown to correlate well-with V_D_/V_T_ (25). Hence, the dead space fraction was calculated by the equation AVDSF = (P_a_CO_2_ – etCO_2_)/P_a_CO_2_. Negative AVDSF values were assumed to be artifactual and were excluded from analysis (26).

Oxygenation index (OI) and oxygenation saturation index (OSI) were used to risk stratify (*mild, moderate*, and *severe*) patients with PARDS in accordance with the PALICC definition (16)—these were calculated on day 2 of PARDS. Admission severity scores were used to benchmark the overall severity of illness of all patients, including the Pediatric Index of Mortality 2 (PIM 2) and the Pediatric Logistic Organ Dysfunction (PELOD) score (20, 21). Multiorgan dysfunction was defined as having at least one extrapulmonary organ dysfunction, regardless of whether it was present before or after PARDS onset (27). The vasoactive inotrope score (VIS) was used to quantify the amount of cardiovascular support, VIS = dopamine dose (μg/kg/min) + dobutamine dose (μg/kg/min) + 100 × epinephrine dose (μg/kg/min) + 10 × milrinone dose (μg/kg/min) + 10,000 × vasopressin dose (unit/kg/min) + 100 × norepinephrine dose (μg/kg/min) (28), and was scored on the same day as PARDS diagnosis. In this study, majority of patients were ventilated on pressure-controlled ventilation and driving pressure was estimated by calculating the peak inspiratory pressure minus end expiratory pressure (without inspiratory hold and paralysis).

### Outcomes and Statistical Analysis

The primary outcome of this study was PICU mortality, with secondary outcomes of multiorgan dysfunction, duration of mechanical ventilation, 28-day ventilator-free days (VFDs), duration of PICU stay, and 28-day PICU-free days (IFDs). Patients were followed up to 28 days or hospital discharge, whichever is later. Continuous and categorical variables were reported as median (interquartile range) and counts (percentages), respectively. As AVDSF was a continuous and dynamic variable, we calculated the mean over the first 7 days of PARDS to obtain a representative index for further analysis. Receiver operating characteristic curves (ROC) were generated to examine relationships between the true-positive rate (sensitivity) and false-positive rate (1—specificity) of AVDSF on mortality, and its optimal predictive cutoffs were determined according to the Youden's index (YI = sensitivity + specificity −1) as the criterion for cutoff. This cutoff was used to classify patients into “high AVDSF” and “low AVDSF” groups. YI is a single statistic that captures the performance of a dichotomous diagnostic test (29). The diagnostic ability of categorical AVDSF was also compared in terms of the area under the curves (AUC). Characteristics and outcomes of patients were compared between AVDSF categories using the Mann–Whitney *U*-test for continuous variables and chi-square for categorical variables. We also compared the prior initial AVDSF (within 24 h of PARDS) cutoff of 0.25 and mean AVDSF 0.25 (17) with the cutoff identified with the ROC and YI analysis in our cohort using sensitivity, specificity, positive predictive value (PPV), and negative predictive value (NPV). Bland–Altman (BA) plot was used for visual representation of the degree of agreement between P_a_CO_2_ and etCO_2_. Coefficient of determination (*R*^2^) was used to determine the strength of association between P_a_CO_2_ and etCO_2_ and between mean AVDSF and admission severity scores (PELOD and PIM2). Since AVDSF, OI, OSI, and ventilation parameters were dynamically linked and obtained daily, we used *R*^2^ to determine the association between AVDSF and OI, OSI, and ventilation parameters using daily data.

Multivariable logistic regression analysis was used to assess the independent association between the high AVDSF group and PICU mortality (*a priori* determined covariates included PIM 2 score and inotrope use). Association from logistic regression was expressed as odds ratio with 95% confidence interval (95% CI). Statistical analysis was performed using SAS version 9.4 software (SAS Institute Inc., Cary, North Carolina, United States) and STATA software, Version 15.1 (StataCorp, College Station, TX). Tests were all two-tailed, with a *p*-value < 0.05 defined as statistically significant.

## Results

Over the study period, there were a total of 1,804 PICU admissions. Of these, 88/1,804 (4.9%) patients fulfilled the PALICC definition for PARDS and consent was obtained for 82/88 (93.2%) patients. Three patients were not intubated, and 10 patients were ventilated on alternative modes of ventilation over the first 7 days of PARDS where etCO_2_ measurements were not possible. The remaining 69 patients were included in this analysis. P_a_CO_2_ and etCO_2_ had good linear correlation (*r* = 0.70; *p* < 0.001) and limits of agreement (Supplementary Figure 1).

**Figure 1:**
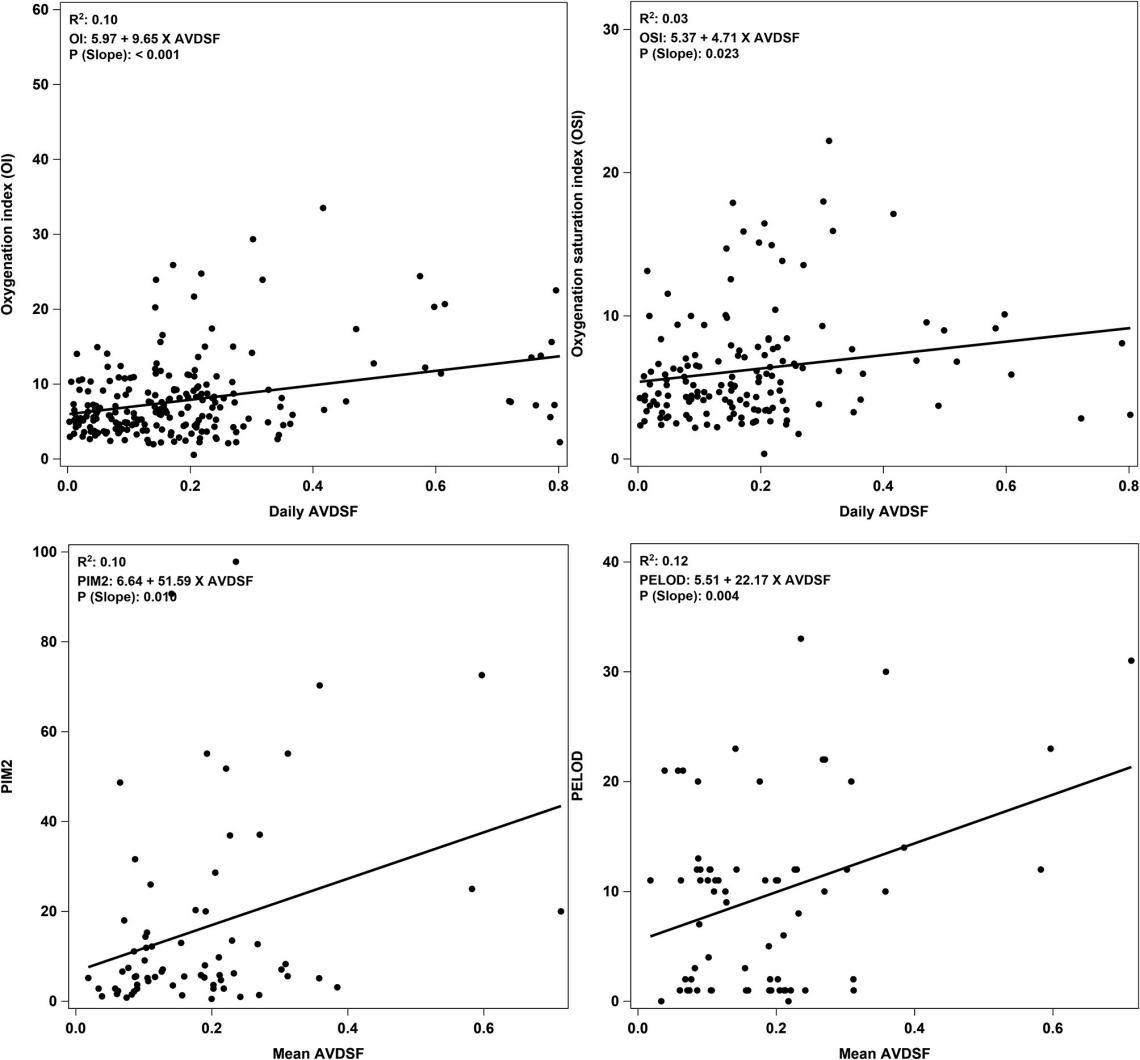
Correlation plots of daily AVDSF with OI and OSI, and mean AVDSF with PIM 2 and PELOD score. Correlation plots for OI and OSI were generated by using daily AVDSF calculations. Correlation plots for PIM2 and PELOD score were generated by using mean AVDSF of 7 days. AVDSF, Alveolar dead space fraction; OI, Oxygenation index; OSI, Oxygen saturation index; PIM 2, Pediatric Index of Mortality 2; PELOD, Pediatric Logistic Organ Dysfunction.

The YI yielded a cutoff of 0.22 and patients with mean AVDSF ≥0.22 were categorized in the “high AVDSF” group, whereas those with mean AVDSF <0.22 were categorized as “low AVDSF.” Younger age [5.1 (1.4, 11.7) vs. 0.9 (0.3, 5.5) years; *p* = 0.0491], the presence of comorbidities [44/50 (88.0%) vs. 10/19 (52.6%); *p* = 0.002], PIM 2 score [5.6 (2.8, 12.2) vs. 13.5 (5.6, 51.8); *p* = 0.016], and PELOD score [7 (1, 11) vs. 12 (8, 22); *p* = 0.008] were significantly associated with higher AVDSF (Table 1). AVDSF correlated with admission PIM 2 (*R*^2^ = 0.10; *p* = 0.010) and PELOD (*R*^2^ = 0.12; *p* = 0.004) scores (Figure 1). Inotropic support [4/50 (8.0%) vs. 9/19 (47.4%); *p* < 0.001] and pulmonary vasodilators [4/50 (8.0%) vs. 9/19 (47.4%); *p* < 0.001] were PICU therapies more often used in the high AVDSF group.

**Table 1:** Clinical characteristic of pediatric acute respiratory distress syndrome patients across high and low AVDSF groups.

**Clinical Characteristics**	**Total (*n* = 69)**	**Low AVDSF (*n* = 50)**	**High AVDSF (*n* = 19)**	***P*-value**
Age, years	4.5 (0.8, 10.6)	5.1 (1.4, 11.7)	0.9 (0.3, 5.5)	0.049
Male gender	42 (60.9)	30 (60.0)	12 (63.2)	0.810
Weight, kg	13.7 (8.2, 30.9)	15.5 (8.9, 36.0)	9.3 (6.4, 14.5)	0.043
Comorbidities present	54 (78.3)	44 (88.0)	10 (52.6)	0.002
Pulmonary PARDS	55 (79.7)	41 (82.0)	14 (73.7)	0.443
PIM2 score	6.6 (3.6, 19)	5.6 (2.8, 12.2)	13.5 (5.6, 51.8)	0.016
PELOD score	10 (1, 12)	7 (1, 11)	12 (8, 22)	0.008
Oxygenation index^*^	9.15 (6.8, 13.4)	6.8 (4.8, 11.3)	10.7 (5.5, 15.0)	0.189
Oxygen saturation index^*^	7.7 (5.8, 10.7)	6.8 (4.8, 8.3)	8.4 (6.9, 11.6)	0.111
High frequency ventilation	23 (33.3)	14 (28.0)	9 (47.4)	0.127
Inotrope	13 (18.8)	4 (8.0)	9 (47.4)	<0.001
VIS	14 (7, 33)	10.0 (5.0, 30.0)	19.5 (10.0, 50.0)	0.194
Pulmonary vasodilator	13 (18.8)	004 (8.0)	09 (47.4)	<0.001
Transfusion	39 (56.5)	25 (50.0)	14 (73.7)	0.076
Systemic corticosteroids	42 (60.9)	31 (62.0)	11 (57.9)	0.755
Neuromuscular blockade	16 (23.2)	10 (20.0)	6 (31.6)	0.309
Prone position	17 (24.6)	13 (26.0)	4 (21.1)	0.670

* *Calculated on day 2 of PARDS.AVDSF, Alveolar dead space fraction; PIM 2, Pediatric Index of Mortality 2; PELOD, Pediatric Logistic Organ Dysfunction; PARDS, Pediatric Acute Respiratory Distress Syndrome; VIS, Vasoactive inotrope score. Continuous and categorical variables described in median (interquartile range) and count (percentage), respectively. p-values are based on Mann–Whitney U-test and chi-square test for continuous and categorical data, respectively*.

**Figure 2:**
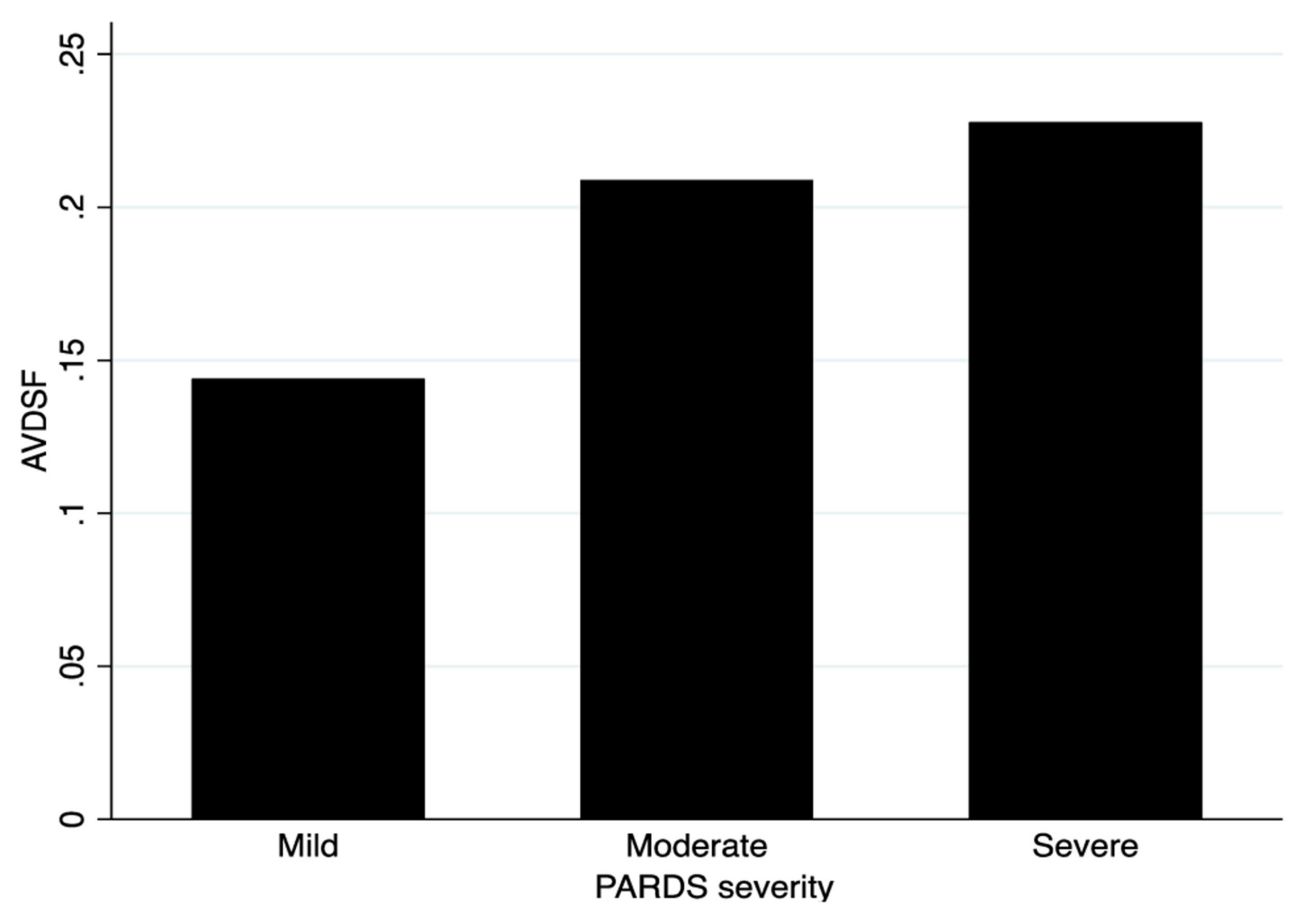
AVDSF across PARDS severity groups. NB: This figure was generated using mean AVDSF of 7 days. AVDSF, Alveolar dead space fraction; PARDS, Pediatric Acute Respiratory Distress Syndrome.

When stratified into PALICC-defined PARDS severity, AVDSF increased with increasing severity, though this did not reach statistical significance [mild 0.14 (0.06, 0.20) vs. moderate 0.21 (0.17, 0.24) vs. severe 0.23 (0.14, 0.36); *p* = 0.053] (Figure 2). Daily AVDSF correlated with daily OI (*R*^2^ = 0.10; *p* < 0.001) and OSI (*R*^2^ = 0.03; *p* = 0.023) (Figure 1) over the first 7 days of PARDS. Higher AVDSF also had a trend toward lower pH and SpO_2_, and higher PaCO_2_, OI, and OSI (Supplementary Figure 2). There was no difference in corresponding daily peak inspiratory pressure, end expiratory pressure, and driving pressure (Supplementary Figure 3) between the high and low AVDSF groups. However, fraction of inspired oxygen and mean airway pressure tended to be higher and tidal volume tended to be lower in the high compared to low AVDSF group.

**Figure 3:**
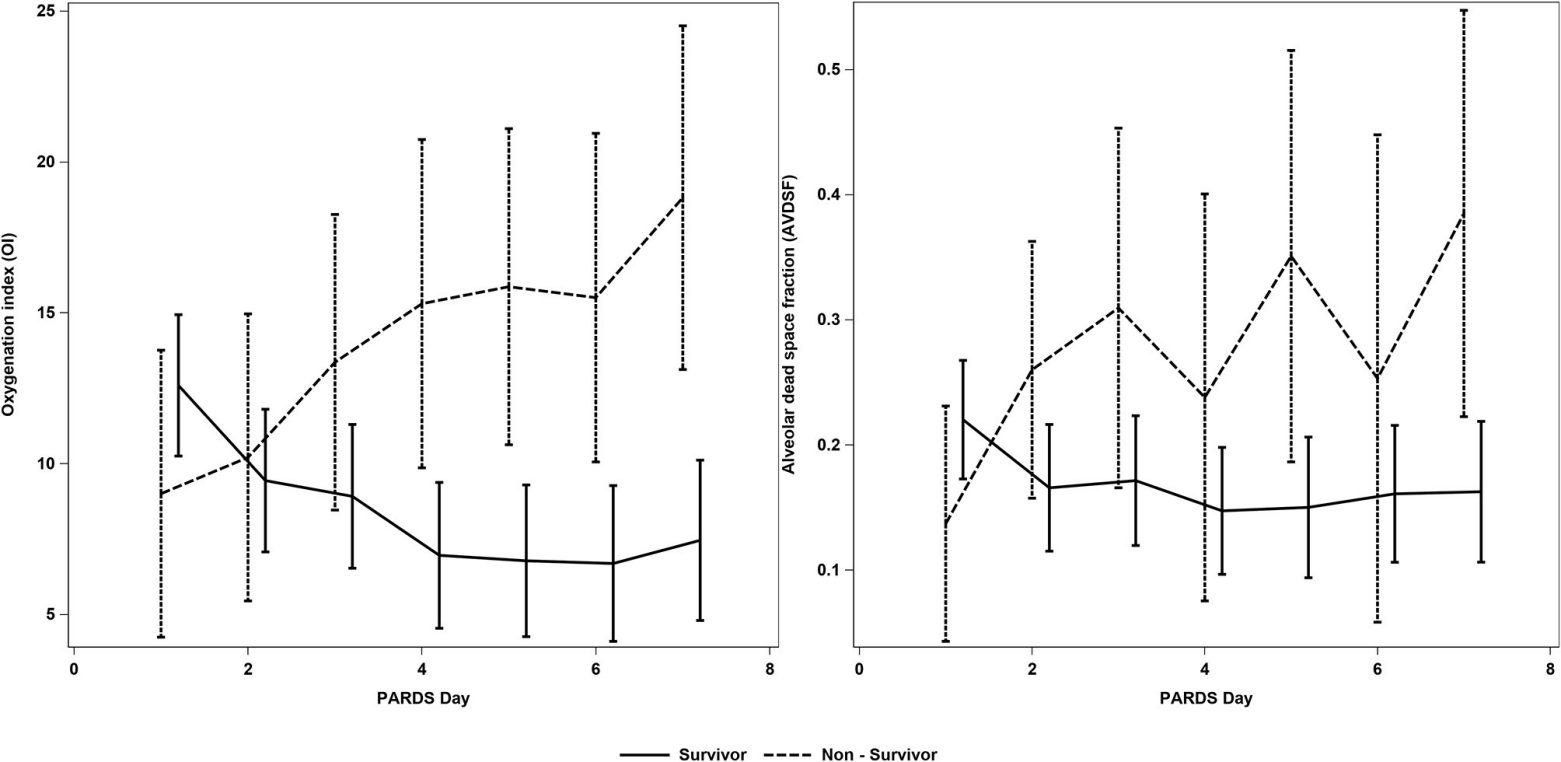
AVDSF trend over the first 7 days of PARDS in survivors and non-survivors. This figure was generated using daily AVDSF and oxygenation index calculations. AVDSF, Alveolar dead space fraction; PARDS, Pediatric Acute Respiratory Distress Syndrome.

Compared to the low AVDSF group, PICU mortality [7/19 (36.8%) vs. 5/50 (10.0%); *p* = 0.009] was higher, and VFD [2 (0, 18) vs. 21 (15, 24); *p* = 0.007] and IFD [0 (0, 16) vs. 16 (5, 21); *p* = 0.013] were lower in the high AVDSF group (Table 2). AVDSF generally trended higher over the first 7 days of PARDS in non-survivors, compared to survivors (Figure 3). This increasing trend was also seen in the OI. In the multivariable model, being in the high AVDSF group [adjusted OR 4.67 (95% CI: 1.12, 19.39)] was significantly associated with mortality (AUC 0.783) (Table 3 and Supplementary Figure 4). The YI and ROC analysis for the outcome of mortality identified mean AVDSF cutoff of 0.22 which had better sensitivity, specificity, and AUC in our cohort compared to initial AVDSF 0.25 from previous studies (17) or mean AVDSF 0.25 (Supplementary Table 3).

**Table 2:** Clinical outcomes across high and low AVDSF groups.

**Outcomes**	**Total (*n* = 69)**	**Low AVDSF (*n* = 50)**	**High AVDSF (*n* = 19)**	***P*-value**
Multiorgan dysfunction^*^	44 (63.8)	30 (60.0)	14 (73.7)	0.291
MV duration, days	7 (4–12)	7 (4, 12)	10 (2, 13)	0.652
VFD, days	19 (2–23)	21 (15, 24)	2 (0, 18)	0.007
PICU duration, days	11 (7–18)	11 (7, 16)	12 (2, 23)	0.830
IFD, days	15 (0–21)	16 (5, 21)	0 (0, 16)	0.013
PICU mortality	12 (17.4)	5 (10.0)	7 (36.8)	0.009

* *Extrapulmonary multiorgan dysfunction. IFD, ICU-free days; MV, Mechanical ventilation; PICU, Pediatric intensive care unit; VFD, Ventilator free days. Continuous and categorical variables described in median (interquartile range) and count (percentage), respectively. p-values are based on Mann–Whitney U-test and chi-square test for continuous and categorical data, respectively*.

**Table 3:** Univariate and multivariable logistic regression for intensive care unit mortality.

**Variables**	**Univariate analysis**		**Multivariable analysis**	
	**Unadjusted OR**	***P*-value**	**Adjusted OR**	***P*-value**
High AVDSF Ref: low	5.25 (1.41, 19.51)	0.013	4.67 (1.12, 19.39)	0.034
PIM 2 score	1.03 (1.01, 1.05)	0.013	1.01 (0.98, 1.04)	0.386
Inotrope Ref: None	3.71 (0.77, 17.93)	0.103	2.28 (0.47, 10.93)	0.304

*AVDSF, Alveolar dead space fraction; PIM 2, Pediatric Index of Mortality 2. Area under the curve (AUC) of this multivariable model is 0.7831*.

## Discussion

This study of AVDSF over the first 7 days of PARDS reveals several important findings. Firstly, day-to-day variations in AVDSF was shown to be closely related to measures of lung injury (OI) and other severity of illness scores (i.e., PIM 2 and PELOD scores). As with the OI, AVDSF seems to increase over the first 7 days in non-survivors, whereas they decreased/remained stable in survivors, indicating that there may be clinical utility in trending AVDSF in patients with PARDS. Secondly, high AVDSF was associated with poor clinical outcomes including higher mortality, and lower VFD and IFD. Lastly, in our cohort, the optimal cutoff AVDSF >0.22 was associated with >4-fold increased odds of mortality. These findings were observed in the context of a cohort of patients with PARDS ventilated on a lung protective mechanical ventilation protocol. AVDSF was expectedly higher in patients on inotropes and pulmonary vasodilators; however, it was indifferent in other therapies thought to improve ventilation/perfusion mismatch (e.g., prone positioning) and bore no association to the end expiratory pressure.

Our study adds to the current literature demonstrating an association between AVDSF and mortality (13, 17, 30) and supports the inclusion of AVDSF into future definitions or risk stratification models for PARDS, along with the current OI and OSI. Prior studies, however, examined AVDSF at onset or at the 24-h mark, while our study examined mean AVDSF over the first 7 days. We also explored the trend of AVDSF and OI over the first 7 days of PARDS illustrating that non-survivors had an increasing AVDSF while survivors had a stable trend, suggesting that there is potentially better prognostic value in trending daily AVDSF (and OI).

AVDSF values differed according to PALICC risk categories in our cohort, with higher values in severe PARDS and lower in the mild PARDS. Physiologically, ventilatory insufficiency increases dead space with no observable effect on oxygenation, whereas circulatory insufficiency may affect both dead space and oxygenation (31). This mechanism accounts for AVDSF differences across PARDS severity groups, along with its correlations with metrics of oxygenation, OI, and OSI. The utility of AVDSF for outcome prognostication or PARDS risk stratification is further supported by its association with other markers of disease severity (e.g., inotrope use, PIM 2, and PELOD scores). General disease severity scores alone, such as Pediatric Risk of Mortality III (PRISM III), PIM2, and PELOD, have been suggested to have poorer performance when used in specific diseases such as PARDS (13). In contrast, OI and OSI only provide pulmonary specific risk stratification. A recent retrospective analysis of 180 pediatric patients with acute hypoxemic respiratory failure found that AVDSF predicted mortality better than OI/OSI (30), with the authors proposing that AVDSF provides information on both pulmonary and extrapulmonary variables. These extrapulmonary variables (such as decreased cardiac output) are in turn associated with mortality (32), consistent with our data.

In evaluating ancillary therapies for PARDS, patients in the high AVDSF group required more inotropic support and pulmonary vasodilators. A common indication for pulmonary vasodilators in patients with PARDS is pulmonary hypertension (16, 33, 34). Pulmonary hypertension in PARDS can be explained by the presence of vasoconstriction and thromboembolism within the pulmonary microcirculation (35)—this is the same pathomechanism that impedes CO_2_ excretion and therefore creates alveolar dead space (12), potentially explaining the association between AVDSF and pulmonary vasodilator use. Though use of pulmonary vasodilators currently does not have demonstrable mortality benefit in PARDS (36), these results suggest that there may be a role for AVDSF to guide its therapeutic use where appropriately indicated. An important caveat would be the discriminatory ability or the cutoff of AVDSF after administration of pulmonary vasodilators; a retrospective analysis of 266 children with PARDS demonstrated that AVDSF at 24 h lost its discriminating ability for mortality after administration of inhaled nitric oxide (AUC 0.64 vs. AUC 0.42), given that AVDSF reflects the status of the pulmonary circulation by the mechanism outlined above (17). Keeping this limitation in mind, AVDSF can still be useful as an easily accessible marker to preliminarily predict various outcomes. Unfortunately, our data were not granular enough to perform a similar analysis on the effects of pulmonary vasodilators. Lastly, AVDSF should theoretically increase with age (or more accurately, height) as airway caliber widens and alveoli grow in numbers and size (37, 38). However, in this study, we observe the opposite effect—this may be confounded by a greater severity of illness in younger patients, though further study is required to validate this finding.

There are several limitations to this study. Firstly, this is a single-center study with a small sample size, thus making it difficult to form any definitive conclusions. Our study found a mortality difference of 25%—using a more conservative difference of 20% between the high and low AVDSF groups, a sample size of 146 patients will be necessary to adequately power (80%) a study. These data may be used for planning future studies. The small event rate (mortality, *n* = 12) also limited our ability to adjust for more potential confounders in the multivariable regression model—as cardiac output may have influenced AVDSF (39), we attempted to account for this in our multivariable model by adding “inotrope” as a covariate. Secondly, etCO_2_ was not measurable when alternative ventilator modes such as HFOV was used—this may have biased the results toward less severe PARDS. Thirdly, though correlation between P_a_CO_2_ and etCO_2_ was shown to be strong and had good limits of agreement, the accuracy of etCO_2_ may still be affected by air leaks. The timing of recording etCO_2_ with corresponding blood gases may also be subject to minor discrepancies. Indeed, 18% of AVDSF calculations in this study yielded negative values, which were assumed to be artifactual and, thus, excluded. We were also not able to analyze all paired etCO_2_ and P_a_CO_2_ for AVDSF calculation throughout the day for the 7 days, which would have provided a more accurate AVDSF value. Lastly, etCO_2_ (and, by extension, AVDSF) only represents alveoli with the longest time constant; this is in contrast to mixed expired partial pressure of CO_2_ measured by volumetric capnography, which accounts for lung heterogeneity (25). Nevertheless, we found etCO_2_ to be a satisfactory and pragmatic surrogate as it is used in routine clinical practice (26, 40).

## Conclusion

AVDSF is associated with increased mortality and decreased VFD and IFD. It correlates with pulmonary indicators of injury (OI), as well as other severity of illness indicators. Our results warrant further evaluation of the usefulness of AVDSF as a marker of disease severity in children with PARDS. As an easily obtainable cardiopulmonary marker, future studies can consider including AVDSF in clinical scores to predict outcomes more robustly in this specific population.

## Data Availability Statement

The raw data supporting the conclusions of this article will be made available by the authors, without undue reservation.

## Ethics Statement

This study involving human participants was reviewed and approved by SingHealth Centralized Institutional Review Board. Written informed consent to participate in this study was provided by the participants' legal guardian/next of kin.

## Author Contributions

JW and HT substantially contributed to the conception and design of the work. AA, HT, Y-JM, and JW were responsible for recruitment of subjects and data collection. RS and JW were responsible for data analysis. RS, JW, and JL were responsible for interpretation of analysis. SO and JW drafted this manuscript. All authors revised it critically for important intellectual content, approved the version to be published, and agree to be accountable for all aspects of the work in ensuring that questions related to the accuracy or integrity of any part of the work are appropriately investigated and resolved.

## Funding

This study was supported by the Pediatric Academic Clinical Program Grant/Tan Cheng Lim Fund (grant number: PAEDSACP-TCL/2020/RES/001) (principal investigator: JW) and the Nurturing Clinician Scientist Scheme (PAEDSACP-NCSS 02/FY2020/P1/14-A29) (principal investigator: JW).

## Conflict of Interest

The authors declare that the research was conducted in the absence of any commercial or financial relationships that could be construed as a potential conflict of interest.

## Publisher's Note

All claims expressed in this article are solely those of the authors and do not necessarily represent those of their affiliated organizations, or those of the publisher, the editors and the reviewers. Any product that may be evaluated in this article, or claim that may be made by its manufacturer, is not guaranteed or endorsed by the publisher.
