# Supplementary material for: The Association Between Alveolar Dead Space Fraction and Mortality in Pediatric Acute Respiratory Distress Syndrome: A Prospective Cohort Study
Source: Front Pediatr. 2022 Feb 28;10:814484. doi: 10.3389/fped.2022.814484 (PMC8918668; doi:10.3389/fped.2022.814484)
Supplement: Supplementary file 1 [file Data_Sheet_1.docx]

**Supplementary Table 1: Lung protective mechanical ventilation protocol**

| **Protocol elements** | **Target patients** | **Targets** |
| --- | --- | --- |
| Peak/ plateau pressure | All CMV | <28 cmH2O |
| Tidal volume | All CMV | 3-6ml/kg |
| Positive end expiratory pressure | All CMV | Incremental FiO2/PEEP combinations:   \| FiO_2_ \| .30 \| .40 \| .40 \| .40 \| .50 \| .50 \| .60 \| \| --- \| --- \| --- \| --- \| --- \| --- \| --- \| --- \| \| PEEP \| 5 \| 6 \| 8 \| 10 \| 10 \| 12 \| 12 \| \| FiO_2_ \| .60 \| .70 \| .70 \| .80 \| .80 \| .90 \| 1.0 \| \| PEEP \| 14 \| 14 \| 16 \| 16 \| 16 \| 16 \| 16 \| |
| Permissive hypercapnia | Moderate & severe PARDS | pH 7.2-7.30 |
| Permissive hypoxia | At risk & mild PARDS | SpO_2_ 92-97% |
|  | Moderate & severe PARDS | SpO_2_ 88-92% |

CMV – conventional mechanical ventilation

PARDS – pediatric acute respiratory distress syndrome

Reference: Wong JJM, Lee SW, Tan HL *et al*. Lung-Protective Mechanical Ventilation Strategies in Pediatric Acute Respiratory Distress Syndrome. Pediatr Crit Care Med 2020; 21(8):720-728.

**Supplementary Table 3: Performance of AVDSF cut-offs for the outcome of mortality**

| **Cut-off** | **Sensitivity (%)** | **Specificity (%)** | **PPV (%)** | **NPV (%)** | **AUC (%)** |
| --- | --- | --- | --- | --- | --- |
| Mean AVDSF 0.22 | 58.3 | 78.9 | 36.8 | 90.0 | 68.8 |
| Mean AVDSF 0.25 | 33.3 | 82.5 | 28.6 | 85.5 | 58.8 |
| Initial* AVDSF 0.25 | 18.2 | 73.1 | 15.4 | 76.9 | 54.3 |

*Initial AVDSF in the first 24hours

AVDSF – Alveolar dead space fraction, PPV – positive predictive value, NPV – negative predictive value, AUV – area under the curve

**Supplementary Figure 1: Correlation plots of partial pressure of arterial carbon dioxide (P_a_CO_2_) with end-tidal carbon dioxide (etCO_2_) (top), and Bland-Altman plot of P_a_CO_2_ with etCO_2_ (bottom)**

NB: These plots were generated using daily etCO_2_ and P_a_CO_2_ measurements

**Supplementary Figure 2: Blood gas and oxygenation indices in high and low AVDSF groups over the first seven days of pediatric acute respiratory distress syndrome**

NB: This figure was generated using daily blood gas and oxygenation indices

AVDSF – Alveolar dead space fraction

P_a_CO_2_ – partial pressure of arterial carbon dioxide

P_a_O_2_ – partial pressure of arterial oxygen

SpO_2_ – pulse oximetry

**Supplementary Figure 3: Ventilator settings in high and low AVDSF groups over the first seven days of pediatric acute respiratory distress syndrome**

NB: This figure was generated using daily ventilator settings

AVDSF – Alveolar dead space fraction

**Supplementary Figure 4: Multivariable receiver operating curve for the outcome of mortality based on AVDSF groups**

NB: This ROC curve was generated by using mean AVDSF of 7 days

AVDSF – Alveolar dead space fraction, ROC – receiver operating curve
