# Supplementary figures and images for: The Association Between Alveolar Dead Space Fraction and Mortality in Pediatric Acute Respiratory Distress Syndrome: A Prospective Cohort Study
Source: Front Pediatr. 2022 Feb 28;10:814484. doi: 10.3389/fped.2022.814484 (PMC8918668; doi:10.3389/fped.2022.814484)

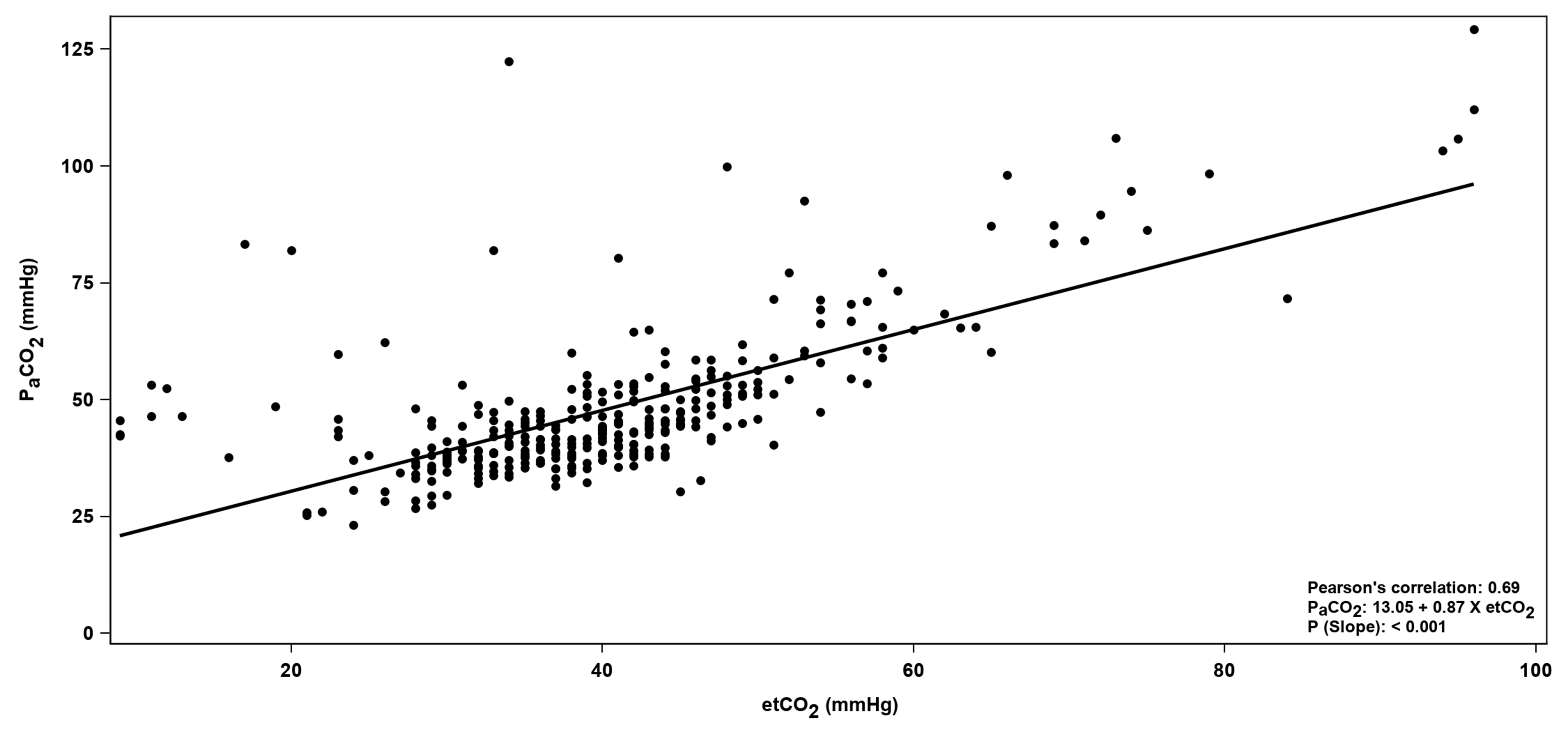

Supplement: Supplementary file 2 [file Image_1.TIF]

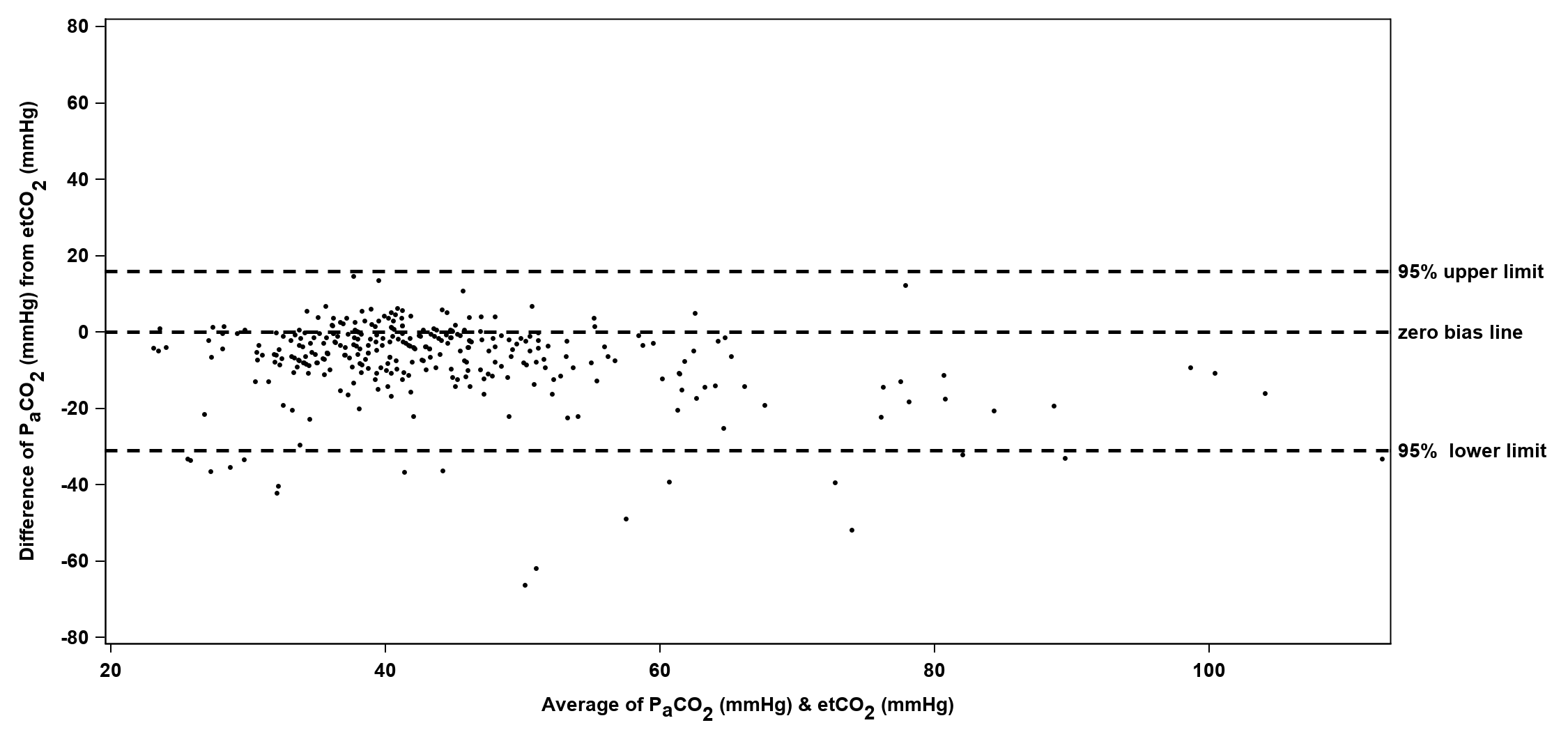

Supplement: Supplementary file 3 [file Image_2.TIF]

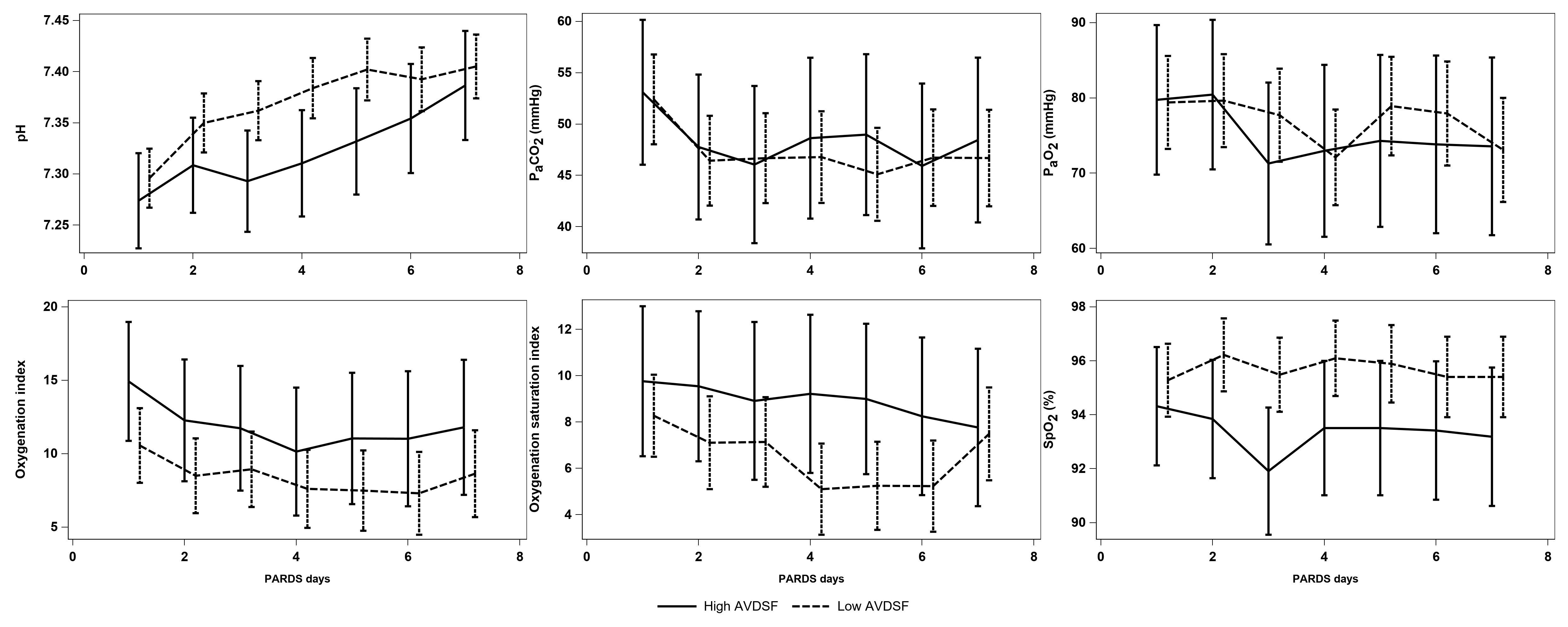

Supplement: Supplementary file 4 [file Image_3.TIF]

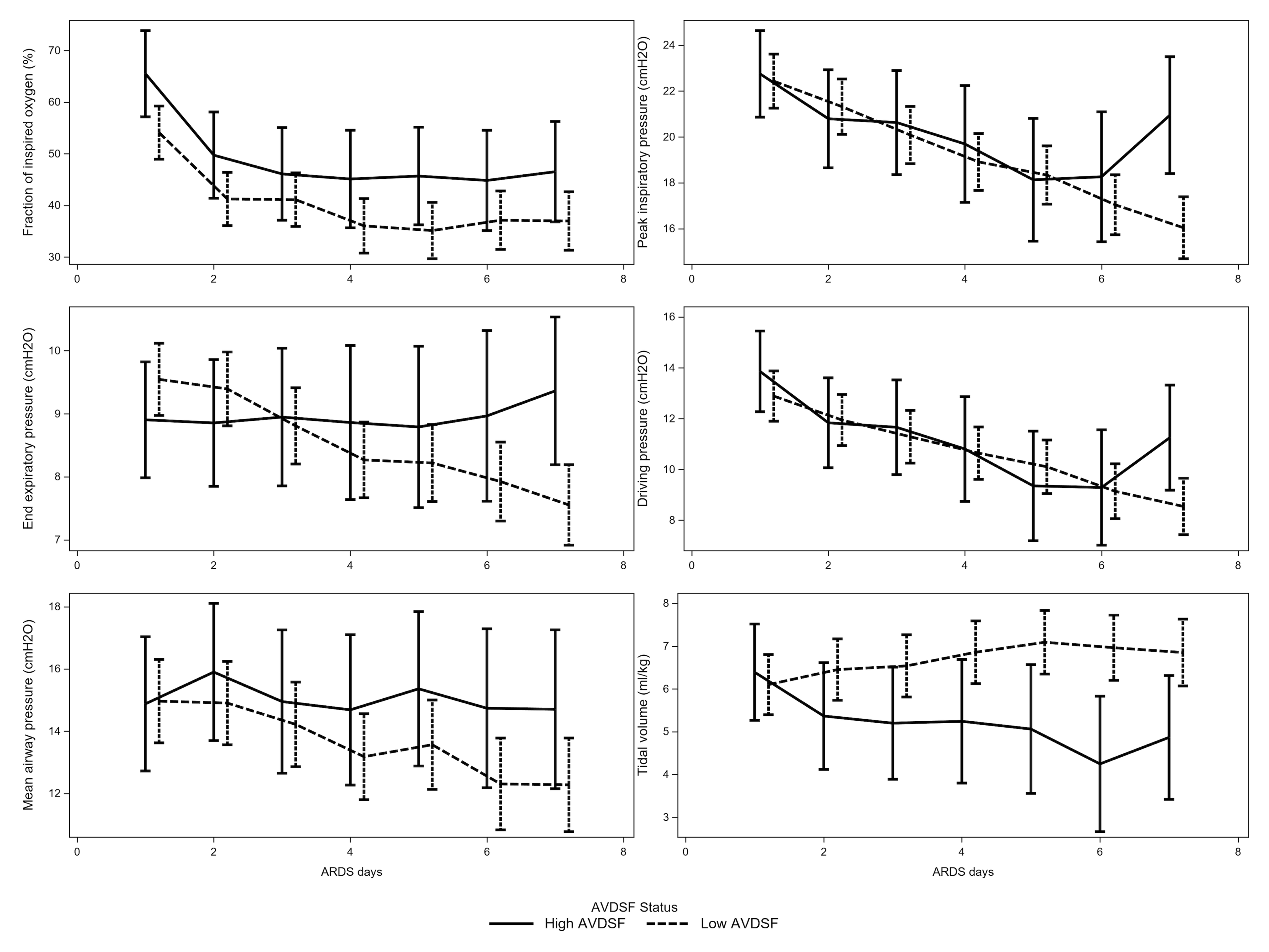

Supplement: Supplementary file 5 [file Image_4.TIF]

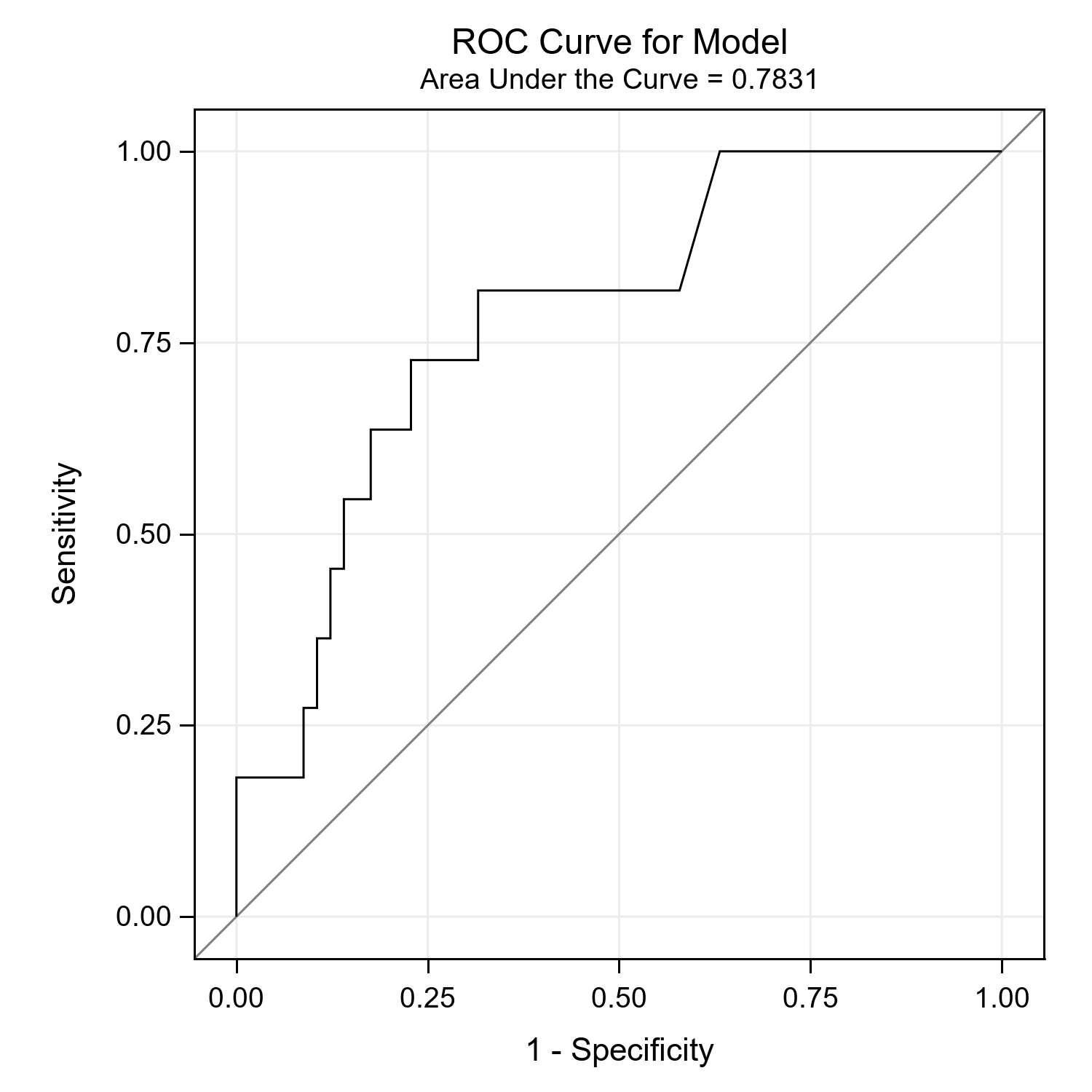

Supplement: Supplementary file 6 [file Image_5.TIF]
